# Core language brain network for fMRI language task used in clinical applications

**DOI:** 10.1162/netn_a_00112

**Published:** 2020-02-01

**Authors:** Qiongge Li, Gino Del Ferraro, Luca Pasquini, Kyung K. Peck, Hernán A. Makse, Andrei I. Holodny

**Affiliations:** Levich Institute and Physics Department, City College of New York, New York, USA; Physics Department, The Graduate Center of City University of New York, New York, USA; Levich Institute and Physics Department, City College of New York, New York, USA; Department of Radiology, Memorial Sloan Kettering Cancer Center, New York, USA; Department of Radiology, Memorial Sloan Kettering Cancer Center, New York, USA; Neuroradiology Unit, NESMOS Department Sant’Andrea Hospital La Sapienza University, Rome, Italy; Department of Radiology, Memorial Sloan Kettering Cancer Center, New York, USA; Department of Medical Physics, Memorial Sloan Kettering Cancer Center, New York, USA; Levich Institute and Physics Department, City College of New York, New York, USA; Department of Radiology, Memorial Sloan Kettering Cancer Center, New York, USA; New York University School of Medicine, New York, USA; Neuroscience, Weill Medical College of Cornell University, New York, USA

**Keywords:** Task-based fMRI, Functional language networks, Healthy controls, Graph theory, Presurgical langugage mapping, *k*-core

## Abstract

Functional magnetic resonance imaging (fMRI) is widely used in clinical applications to highlight brain areas involved in specific cognitive processes. Brain impairments, such as tumors, suppress the fMRI activation of the anatomical areas they invade and, thus, brain-damaged functional networks present missing links/areas of activation. The identification of the missing circuitry components is of crucial importance to estimate the damage extent. The study of functional networks associated with clinical tasks but performed by healthy individuals becomes, therefore, of paramount concern. These “healthy” networks can, indeed, be used as control networks for clinical studies. In this work we investigate the functional architecture of 20 healthy individuals performing a language task designed for clinical purposes. We unveil a common architecture persistent across all subjects under study, that we call “core” network, which involves Broca’s area, Wernicke’s area, the premotor area, and the pre-supplementary motor area. We study the connectivity of this circuitry by using the *k*-core centrality measure, and we find that three of these areas belong to the most robust structure of the functional language network for the specific task under study. Our results provide useful insights on primarily important functional connections.

## Author Summary

Neurosurgeons employ language fMRI to localize important language areas for patients with brain impairment. Yet, brain pathologies (e.g., brain tumors, strokes, epilepsy) affect functional connectivity by disrupting functional links and suppressing the activation of brain areas. Thus, although clinical tasks are designed to guarantee robust activation, the functional connectivity of patients with brain pathologies is ultimately damaged by brain impairments. To better quantify the damage produced by the brain pathology on the functional connectivity, it is paramount to have, as a benchmark, functional networks of healthy individuals who perform a task for clinical cases. Our findings identify a group of functional regions of interest linked together in a functional circuitry that have a decisive role for the language task used in clinical applications.

## INTRODUCTION

Broca’s area (BA) and Wernicke’s area (WA) have long been recognized as essential language centers. Studies of aphasic patients have shown that damage to BA and WA causes loss of ability to produce speech (expressive aphasia) and difficulty understanding language (receptive aphasia), respectively (Dronkers, Plaisant, Iba-Zizen, & Cabanis, 2007; Wernicke, 1970). Further evidence has shown that other secondary and tertiary anatomical brain areas are also involved in language Friederici (2011), including the pre-supplementary motor area (pre-SMA; Hertrich, Dietrich, & Ackermann, 2016), the premotor area (preMA; Duffau et al., 2003), and the basal ganglia (Booth, Wood, Lu, Houk, & Bitan, 2007). Despite this evidence, a full characterization of the language network is still debated (Friederici, Chomsky, Berwick, Moro, & Bolhuis, 2017; Fedorenko & Kanwisher, 2009).

Functional MRI (fMRI) has been largely used to investigate the blood-oxygen-level dependent (BOLD) activation of the human brain, for both clinical and research purposes. Although it cannot fully resolve the issue of “functional specialization” of brain regions by itself, it sheds light on which regions are engaged in certain cognitive processes. Therefore fMRI allows us to constrain hypotheses on the structure of the language network.

Language has been investigated using both resting-state fMRI (rs-fMRI) and tasked-based fMRI (tb-fMRI). The former studies brain activation of subjects at rest (Lee, Smyser, & Shimony, 2013), whereas tb-fMRI delineates brain areas functionally involved in the performance of a specific task (Bookheimer, 2002). Task-based fMRI is task-dependent, that is, different language tasks may activate different areas involved in language function (Xiong et al., 2000). Consequently, clinical studies employ a specific class of language tasks that have been shown to produce robust activation in individual participants and thus facilitate the localization of the language-sensitive cortex (Brennan et al., 2007; Ramsey, Sommer, Rutten, & Kahn, 2001).

In this paper we analyze fMRI scans of 20 healthy individuals who perform the same language task designed for clinical purposes. From the correlation of the BOLD signal we construct the functional connectivity network for each subject, which is standardly employed to investigate statistical interdependencies among brain regions (Bullmore & Sporns, 2009; Hermundstad et al., 2013; Gallos, Makse, & Sigman, 2012). We then employ graph theory to study the networks’ properties as successfully done in Del Ferraro et al. (2018) to investigate memory formation. The motivation for this study is to use the resulting functional connectivity of these healthy individuals as a benchmark for clinical study, as we explain next. We employ a language clinical task because we are interested in studying the fMRI activation associated with this specific type of task. We employ healthy subjects and not patients with brain impairments because we want to study the fMRI activation without any interference that might arise because of the brain impairment. Brain pathologies (e.g., brain tumors, Wang et al., 2013; strokes, Tombari et al., 2004; epilepsy, Rosenberger et al., 2009) indeed affect functional connectivity by disrupting functional links and reducing the fMRI activation of brain areas (e.g., the neurovascular decoupling effect due to brain tumor; Aubert, Costalat, Duffau, & Benali, 2002). The reconstruction of the functional connectivity in clinical cases, therefore, is influenced by the presence of brain pathology (Wang et al., 2013). In other words, the functional connectivity of a patient with a pathology such as a brain tumor, for instance, presents missing links and missing fMRI active areas compared with the healthy case, for the same specific task. To better understand what functional damage was produced by the brain impairment, it is important to have, as a benchmark, functional networks of healthy individuals performing the same language task normally used for clinical cases. In this way, using clinical language tasks performed by healthy subjects, we can study functional networks associated with clinical tasks without perturbations that arise from brain impairments. These functional networks can be used as benchmarks for other studies that use the same type of task but employ patients with brain damage. The comparison between a healthy control and a patient’s functional network relative to the same task could in principle establish what is the damage produced by the brain impairment on the functional network and might, among others, guide tumor resection to preserve functional links.

Motivated by these considerations, we investigated which is the language functional architecture shared among healthy subjects, that is, the functional subnetwork that persists in each analyzed individual beyond the intersubject variability. This architecture is indicative of a core structure for the language task under study shared across individuals. Core architectures have been identified in other contexts (Bassett et al., 2013), but very little is known about the core for language tasks (Chai, Mattar, Blank, Fedorenko, & Bassett, 2016) and its investigation is one of the main goals of our study.

Furthermore, we aim to uncover the functional connectivity of the subdivisions of the Broca’s area (pars-opercularis, op-BA, and pars-triangularis, tri-BA, i.e., Brodmann area 44 and 45 respectively), which plays a pivotal role in language function (Dronkers et al., 2007; Friederici, 2011). Previous studies based on fMRI showed that BA’s subdivisions perform different functions in language processing. Newman, Just, Keller, Roth, and Carpenter (2003) showed that tri-BA is more implicated in thematic processing whereas op-BA is more involved in syntactic processing. Studies based on transcranial magnetic stimulation have shown that op-BA is more specialized in phonological tasks and tri-BA more in semantic tasks (Devlin, Matthews, & Rushworth, 2003; Gough, Nobre, & Devlin, 2005; Nixon, Lazarova, Hodinott-Hill, Gough, & Passingham, 2004). Patients who show speech impairment often have direct damage to the Broca’s area. Thus, understanding how BA subdivisions are functionally wired to other brain regions in healthy controls may be used for comparison in some clinical cases and could potentially be of help to better clarify the effect of brain pathologies on this decisive language area.

From our analysis we find that the functional architecture shared by most of the subjects under study wires together Broca’s area (op-BA and tri-BA), Wernicke’s area, the pre-supplementary motor area, and the premotor area. By investigating network properties at the subject level we find that, in each individual functional network, these areas belong to an innermost core, more specifically the maximum *k*-core of the functional connectivity, which is a robust and highly connected substructure of the functional architecture. The *k*-core measure has received vast attention in network analysis since it provides a topological notion of the structural skeleton of a circuitry (Kitsak et al., 2010; Pittel, Spencer, & Wormald, 1996; Rubinov & Sporns, 2010; Dorogovtsev, Goltsev, & Mendes, 2006). More recently, the maximum *k*-core has been related to the stability of complex biological systems (Morone, Del Ferraro, & Makse, 2019) and of resilient functional structures in the brain (Lucini, Del Ferraro, Sigman, & Makse, 2019). Our results demonstrate that the functional architecture that persists beyond intersubject variability is part of the maximum *k*-core structure, an innermost highly connected subnetwork, associated with a system’s resilience and stability (Morone et al., 2019).

Overall, our findings identify a group of functional regions of interest (fROIs) linked together in a functional circuitry that play a decisive role for the language task used in clinical applications.

## MATERIALS AND METHODS

The study was approved by the Institutional Review Board and an informed consent was obtained from each subject. The study was carried out according to the declaration of Helsinki. Twenty healthy right-handed adult subjects (13 males and 7 females; age range 36 years, mean = 36.6; *SD* = 11.56) without any neurological history were included.

### Functional Task

**Figure 1.**
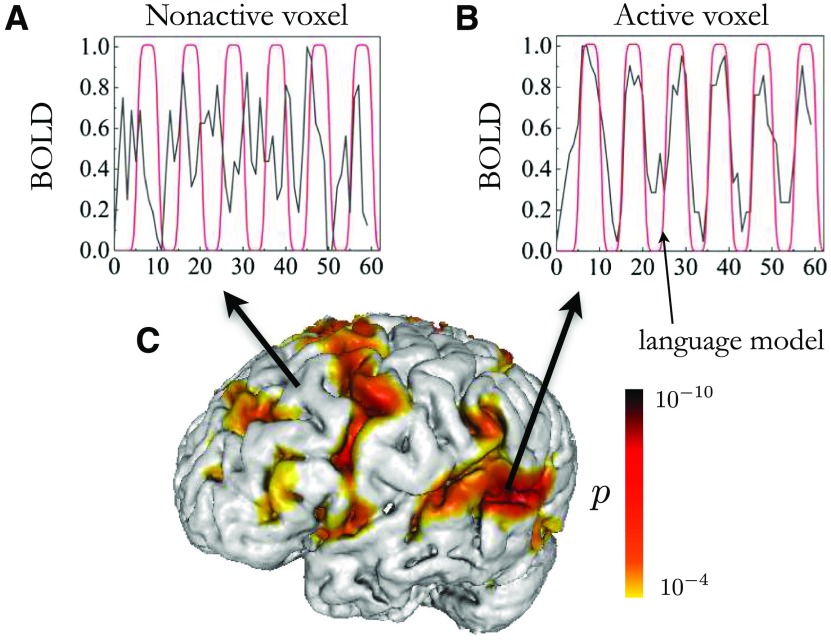
Activation map for a representative subject. BOLD signal for a nonactive and active voxel are shown respectively in panels A and B together with the smoothed boxcar language model, which depicts the auditory stimulus. The black curve represents BOLD signal, while the red curve represents the smoothed boxcar task model design. The red curve’s peaks represent the continuous stimuli (verb task), presented for a certain period of time (10 s), the “task” or “on” period. The red curve’s troughs represent the rest period (no task) for the participant, which lasted 10 s, (C) 3D visualization of the brain with fMRI active areas and corresponding *p* values.

For the fMRI task, all subjects performed a verbal fluency task using verb generation in response to auditory nouns. During the verb generation task, subjects were presented with a noun (for example, baby) by oral instruction and then asked to generate action words (for example, cry, crawl) associated with the noun. Four nouns were displayed over six stimulation epochs, with each epoch lasting 20 s, which allowed for a total of 24 distinct nouns to be read over the entire duration. Each epoch consisted of a resting period and a task period (see BOLD activation in Figures 1A and1B). In order to avoid artifacts from jaw movements, subjects were asked to silently generate the words. Brain activity and head motion were monitored using Brainwave software (GE, Brainwave RT, Medical Numerics, Germantown, MD), allowing real-time observation.

### Data Acquisition

A GE 3T scanner (General Electric, Milwaukee, Wisconsin, USA) and a standard quadrature head coil was employed to acquire the MR images. Functional images covering the whole brain were acquired using a T2*-weighted gradient echo planar imaging sequence (repetition time, TR/echo time, TE = 4,000/40 ms; slice thickness = 4.5 mm; matrix = 128 × 128; FOV = 240 mm). Functional matching axial T1-weighted images (TR/TE = 600/8 ms; slice thickness = 4.5 mm) were acquired for anatomical coregistration purposes. Additionally, 3D T1-weighted SPGR (spoiled gradient recalled) sequences (TR/TE = 22/4 ms; slice thickness = 1.5 mm; matrix = 256 × 256) covering the entire brain were acquired.

### Data Processing

Functional MRI data were processed and analyzed using the software program Analysis of Functional NeuroImages (AFNI; Cox, 1996). Head motion correction was performed using 3D rigid-body registration. The first volume was selected to register all other volumes. The first volume was chosen because it was acquired right next to the anatomical scan. During the registration, the motion profile was saved and during the statistical analysis any voxels highly correlated with the profile were regressed out. Spatial smoothing was applied to improve the signal-to-noise ratio using a Gaussian filter with 4 mm full width of half maximum. Corrections for linear trend and high-frequency noise were also applied. To obtain the activation map, the rectangular train representing the single-task (verb generation) block design is convolved with a canonical hemodynamic response function (HRF; see the red curve in Figures 1A and 1B). This time series is one regressor in the general linear model where the preprocessed BOLD response is to fit to this as well as a baseline (intercept). The BOLD signal is represented by the black curve in Figures 1A and 1B). The test statistics for the activation maps are determined by cross-correlation analysis within AFNI software. They were generated in the individual native space at a minimum threshold of *p* < 0.0001 to identify activated voxels set by a neuroradiologist (see Figure 1C, for a representative subject).

The individual voxel threshold level (uncorrected *p* < 0.0001) was set to minimize the contribution of false positives that could be caused by stimulus-correlated head motion and/or random noise fluctuation. The level was set for all subjects. We looked at FDR (*q* value calculated in AFNI software) to control false positives among the detected voxels, and the FDR corrected is *q* < 0.001. We verified that the activated areas identified and considered in our graph theory analysis are significant.

### Network Construction

The following sections describe the functional network construction. In the first subsection, we first describe how to create, from the fMRI signal of the active voxels, a brain network for each individual separately. The second subsection discusses the group analysis or how we obtain, from the individual brain networks, a common architecture that unveils a persistent circuitry across all the single-subject brain networks.

##### Individual brain network construction.

For each subject we construct a functional network. This network can be seen at two different scales or levels: (a) at the *voxel level* and (b) at the *fROI level*, as we explain in more detail below.

At the **voxel level**, active voxels in the individual activation map (*p* < 0.0001) define the nodes of our functional network, where a voxel is the lowest resolution measured by fMRI. Functional links are inferred by thresholding pairwise Pearson correlations (see Equation 1) between a pair of voxels, as standard in the literature (Bullmore & Sporns, 2009; Gallos et al., 2012; Hermundstad et al., 2013).

The pairwise correlation is defined as follows:

$$C_{ij}=\frac{\langle x_{i}x_{j}\rangle -\langle x_{i}\rangle \langle x_{j}\rangle }{\sqrt{{\left(\langle x_{i}^{2}\rangle -\langle x_{i}\rangle \right)}^{2}{\left(\langle x_{j}^{2}\rangle -\langle x_{j}\rangle \right)}^{2}}},$$ (1)

where *x*_*i*_ is a vector encoding the fMRI time response of voxel *i* and 〈⋅〉 indicates a temporal average.

**Figure 2.**
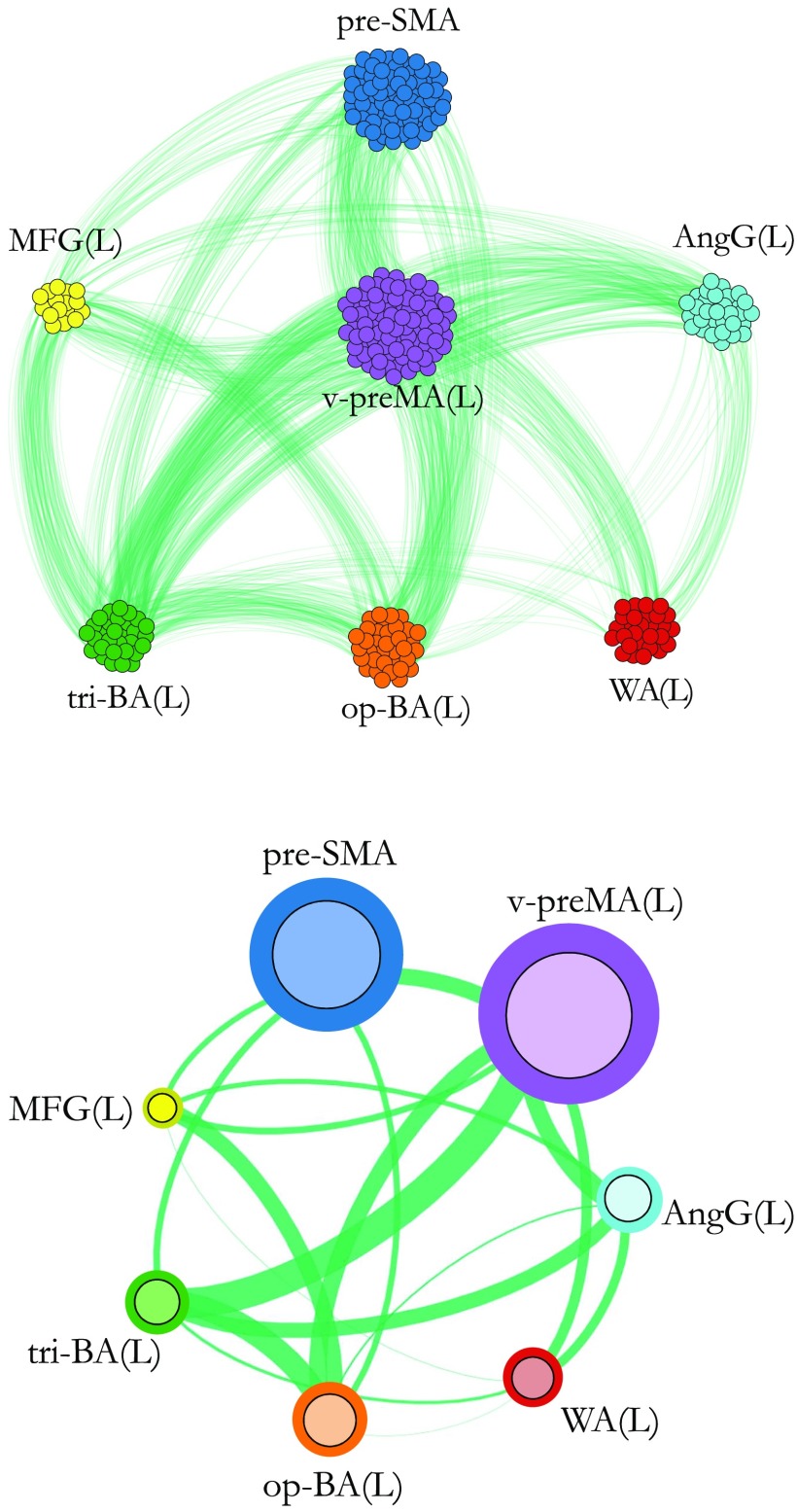
Two visualizations of the individual functional network. The figure shows functional networks for a representative subject relative to the fMRI active brain areas during the language task. Upper panel: voxel-level network. Each node in the network represents a voxel, each link connects a pair of voxels in different brain modules, and it is indicative of functional interdependency. Links connecting voxels within the same brain module are not visible but exist. Lower panel: fROI-level network for the same voxel-level architecture shown in the upper panel. Voxels belonging to the same anatomical region are grouped into an fROI, represented as a node in the network. Node’s size is proportional to the number of voxels in the fROI. Colored borders have no meaning and are used only for illustrative purpose. Each link’s thickness connecting two fROIs is proportional to the sum of link’s weight connecting all the voxels in the two fROI (exact definition given in Equation 2).

Accordingly, pairs of voxels with correlation above a fixed threshold are connected by a link (Bullmore & Sporns, 2009; Del Ferraro et al., 2018; Lucini et al., 2019). The threshold is an absolute threshold, which means we pick a reference value of the correlation as the smallest and assign a zero to any value below. The link weight is given by the correlation strength as defined above in Equation 1. Nearby active voxels are grouped together based on each subject’s individual anatomy and are considered part of the same fROI. Figure 2, upper panel, shows a realization of the voxel-level functional network for a representative subject, where voxels that are part of the same fROI are colored equally.

We define fROIs within each subject individually, based on the activation and anatomy of the specific subject (Lucini et al., 2019; Del Ferraro et al., 2018; Fedorenko, Hsieh, Nieto-Castañón, Whitfield-Gabrieli, & Kanwisher, 2010). For instance, all the active voxels in Brodmann area 22 of the superior temporal lobe define the Wernicke’s area fROI. The reason for choosing individual-based fROIs is that group-based ROI-level analysis suffers from intersubject variability in the location of activation. In contrast, individual-subject-based fROI analysis can reveal greater functional specificity (Fedorenko et al., 2010). Furthermore, working in individual native space prevents the propagation of errors due to coregistration to universal ATLAS.

At the **fROI level or module level** a node represents an entire fROI, that is, a group (collection) of nearby active voxels in the spatially (anatomically) proximate area. Hereafter, we might use the word “module” or brain “region” as substitute of the word fROI. At this level, a functional link connects two fROIs if and only if there exists at least one link, at the voxel level, between a pair of voxels in the two fROIs. The functional link’s weight between two fROIs *i* and *j* (*W*_*ij*_) is defined as the sum of the number of links connecting pairs of voxels between the two fROIs, normalized by the sum of the two fROIs’ size:

$$W_{ij}=\frac{\text{\#links}\;\text{connecting}\;i↔j}{\text{size}\;({\text{fROI}}_{i})+\text{size}\;({\text{fROI}}_{j})}.$$ (2)

For each individual, we then normalize each*W*_*ij*_ by the value of the largest *W* for that individual (*W*^max^):

$${\tilde{W}}_{ij}=\frac{W_{ij}}{W^{\;\text{max}\;}},\;\text{for all i and j fROIs}.$$ (3)

In this way the link’s weight scale is the same across subjects (see Supplementary Table S1) and the maximum weight is $\tilde{W}=1$ in each individual. Figure 2, lower panel, illustrates the functional network at the fROI level for a representative subject.

For each individual, we are interested in uncovering the functional architecture of the subdivision of the Broca’s area, that is, tri-BA and op-BA, which correspond to all active voxels in Brodmann area 44 and 45, respectively. Each of these subareas has been associated with different language processes in previous studies (Devlin et al., 2003; Gough et al., 2005; Newman et al., 2003; Nixon et al., 2004). Through our analysis we aim to find out the specificity of their functional connectivity, to unveil whether their different engagement in language processing may be associated with a different functional wiring with the rest of the brain. Thus, when building the individual functional network, we group the active voxels of the BA into two different and separate fROIs: op-BA and tri-BA (see Figure 2).

We named the fROIs according to their main anatomical boundaries as follows. We retained the classical designations of BA (Brodmann area 44–45, inferior frontal gyrus) and WA (Brodmann area 22, superior temporal gyrus), as these designations still predominate in neurosurgery, which dominates clinical practice (Friederici, 2011). We defined the ventral premotor area (v-preMA) as the ventral portion of the premotor cortex, which includes the inferior part of Brodmann area 6, centered on the posteriormost portion of the middle frontal gyrus (MFG; Friederici, 2011). The superior portion of Brodmann area 6 was considered dorsal premotor area (d-preMA). The anteriormost part of the middle frontal gyrus was identified as anterior middle frontal gyrus (aMFG). The pre-SMA was defined within the medial frontal cortex, at the level of Brodmann area 6 (Nachev, Kennard, & Husain, 2008). The precentral gyrus was identified with Brodmann area 4, the supramarginal gyrus was identified with Brodmann area 40 and angular gyrus with Brodmann area 39 (Friederici, 2011). The deep opercular cortex (DOC) included the innermost portion of the frontal operculum (Friederici, 2011).

The visual and the auditory cortex, which are active areas that support nonlinguistic processing, were excluded from the analysis (Fedorenko et al., 2010; Fedorenko & Kanwisher, 2009). These areas are indeed activated because the subject is presented with auditory stimuli and may keep the eyes open.

The same functional network construction as described above is carried over for all 20 subjects individually, both at the voxel and the fROI level. Next, we carry out a group analysis to identify the common functional network shared across individuals, beyond the intersubject variability, as described in following section.

##### Common network construction across subjects.

Our interest in studying functional networks for single individuals performing language tasks is aimed at uncovering functional architectures that are persistent across healthy subjects and could be useful and informative when dealing with clinical cases. Individual functional networks have innate subject variability (e.g., one subject activates in one specific area or has a functional link while another does not). Therefore, after we reconstructed the individual functional networks, we performed a group analysis at the fROI level by investigating which set of links and brain areas is persistent across subjects or, in other words, which functional subarchitecture is common among all the individuals.

This functional architecture is informative of which areas and functional links persist beyond the intersubject variability, and therefore it represents a language core structure for the specific language task under study. Accordingly, surgical intervention, as for instance tumor resection, should operate by preserving such core structure existing across healthy controls. In addition, functional damage to this structure due to brain pathologies—and observed from the functional connectivity of the patient—may be informative of the damage extent (e.g., a missing functional link in the core may signify a larger harm than a missing connection between more peripheral areas not in the core). We name this most persistent functional architecture across subjects, at the fROI level, *common network*. This common architecture is defined retaining a pair of fROIs and a functional link connecting them only if these areas and link are present across subjects.

The weight of the functional link connecting two fROIs *i* and *j* in the common network ($W_{ij}^{\text{C}}$) is defined as the average of the ${\tilde{W}}_{ij}$ connecting those fROIs across subjects:

$$W_{ij}^{C}=\frac{1}{N}\overset{N}{\underset{l=1}{\sum }}{\tilde{W}}_{ij}^{\left(l\right)},$$ (4)

where *N* is the total number of individuals.

We report and discuss the results of this quantitative analysis in the following sections.

## RESULTS

### Individual Networks

For each individual we observe fMRI activation in both hemispheres, however, left dominance is clearly observed, as expected since all the subjects are right-handed (Isaacs, Barr, Nelson, & Devinsky, 2006; Knecht et al., 2000). The number of left hemisphere areas of activation is greater and in most cases their frequency of activation is greater as well.

Active fROIs across subjects include the following, in alphabetic order: angular gyrus (L), Broca’s area (L; op-BA and tri-BA), Broca’s area (R), caudate (L and R), deep opercular cortex (L and R; Friederici, 2011), aMFG (L and R), precentral gyrus (L and R), ventral and dorsal preMA (L), ventral preMA (R), pre-SMA, supramarginal gyrus (L and R), and Wernicke’s area (L and R). Detailed information on the frequency of activation of each area across subjects is summarized in Supporting Information Table S2. In the following, for brevity, we will refer to left hemisphere brain areas simply with the name of the areas, omitting the specification (L).

The functional network for a representative subject at both the voxel and the fROI level is shown in Figure 2. All the single subjects’ functional connectivity at the fROI level for each of the 20 healthy individuals considered in our study are shown in Supporting Information Figure S1, and all the connectivity values between pairs of fROIs are reported in Supporting Information Table S1.

We observe that, overall, the preMA is the most connected area across subjects, in terms of connectivity weight. In 8 over 20 individuals the strongest functional connection is between preMA–op-BA and in 7 over 20 cases it is between preMA–pre-SMA. In total, the preMA turns out to have the strongest connection with one of the other areas in 17 out of 20 subjects; in 3 cases the strongest functional connection is between op-BA and tri-BA. For further details on the connectivity of each area, see Supporting Information Table S1.

Wernicke’s area is known to structurally connect to BA through the arcuate fasciculus, a bundle of axons linking the inferior frontal gyrus with the superior temporal gyrus. We investigated the functional connections of the BA subdivisions with the rest of the brain and, with focus on WA, at the fROI level, we compared how frequent op-BA connects to WA versus how frequent tri-BA connects to WA. We find that op-BA connects to WA in 18 out of 20 subjects (90% of the cases), while tri-BA connects to WA in 15 out of 20 individuals (75% of the cases). In terms of connectivity weight, in 10 out of 20 subjects (50%) WA connects more strongly to op-BA than to tri-BA, whereas in 7 subjects (35%) we have the opposite finding, tri-BA connects more to WA than the opercular counterpart. In 2 individuals the functional connectivity of op-BA and tri-BA to WA is, instead, approximately the same. One subject does not show WA activation at all.

Regarding other relevant areas such as preMA and pre-SMA, we find that the connectivity frequency of these areas with op-BA and tri-BA is about the same. Indeed the preMA connects to op-BA in 18 subjects and to tri-BA in 17 out of 20. The pre-SMA connects to op-BA in 19 subjects and to tri-BA in 18 individuals. So, overall, the connectivity frequency of the BA subdivisions with preMA and pre-SMA is similar. In terms of connectivity weight, op-BA connects more strongly to both preMA and pre-SMA compared with tri-BA. Thus, although the BA subdivisions connect to preMA and pre-SMA with about the same frequency across subjects, op-BA has, overall, a larger connectivity weight.

**Figure 3.**
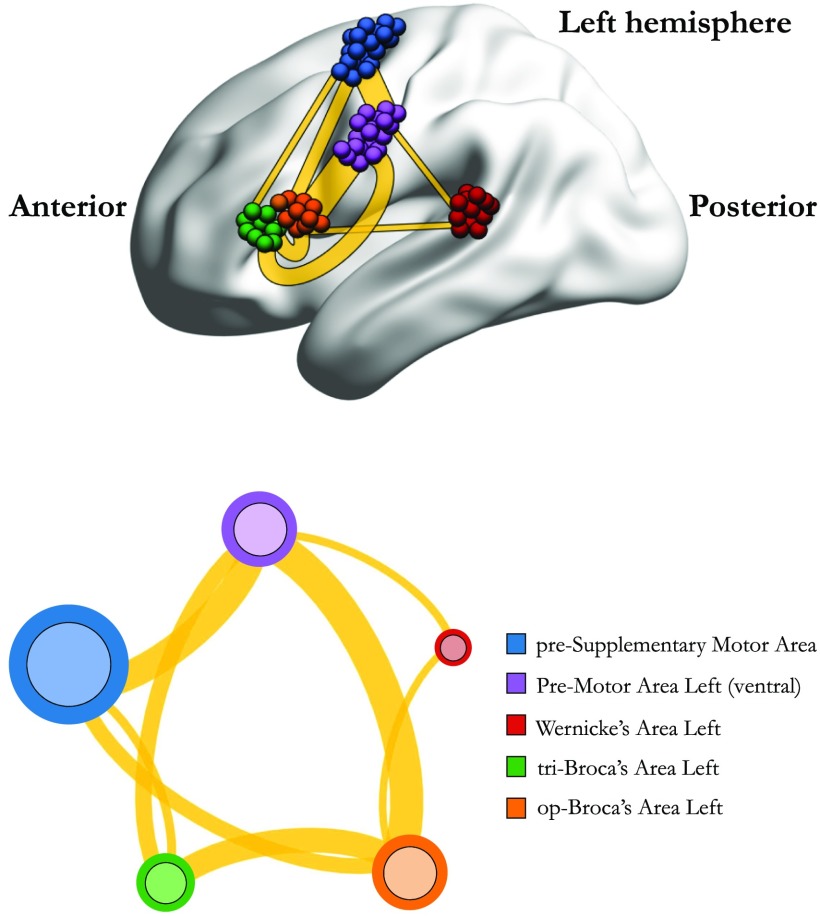
Common network across subjects for the language task under study. The figure illustrates the functional network, beyond intersubject variability, shared across individuals (17 out of 20). The weight of a link connecting two fROIs is proportional to the average of the functional links connecting those fROIs across subjects. Upper panel: fROIs are located on their anatomical location on the brain. Lower panel: pictorial illustration of the network in the upper panel, with the fROIs equally spaced on a plane.

To investigate the overall functional connectivity that each fROI has with the rest of the common network shown in Figure 3 we computed, for each individual, the sum of the functional links of each fROI with the rest of the network. In each subject, each fROI that appears in the common network can then be ranked according to this connectivity strength (most connected at the top and least connected at the bottom). We present these results in Supporting Information Figure S2. The results show that preMA is either the most connected or the second most connected area in 95% of the cases. On the contrary, WA is either the least connected or the second least connected area in 95% of the cases.

### Common Network Across Subjects and Functional Subdivisions of Broca’s Area

The common network at the fROI level, as described in the Common Network Construction section, is made by those fROIs and links present (persistent) across the majority of subjects. As a result of the left dominance at the individual level, no consistent overlap of right-hemisphere activation has been found across subjects.

We find that the persistent structure across individuals (17 over 20), beyond intersubject variability, is made by op-BA, tri-BA, WA, preMA, and pre-SMA connected together in a functional architecture (see Figure 3). This circuitry represents the core structure for the specific clinical language task under investigation since it is the functional architecture that prevails in nearly all subjects. We find this network in 17 over 20 subjects and not in all of them because three subjects show lack of activation for either the op-BA (1 case), the tri-BA (1 case), or neither WA nor tri-BA (1 case). The common network shown in Figure 3 is therefore the one prevailing in closely all the subjects and, thus, the functional structure that is persistent beyond intersubject variability. We tested the robustness of these results by varying the threshold in each individual network (discussed in the Individual Brain Network Construction section) by 5−10% and by recomputing the common network analysis. We find that within this range of variation the common network is made by the same brain modules (fROIs) and links shown in Figure 3.

Furthermore, this conclusion is additionally supported by additional findings we obtained on a study conducted on bilingual healthy subjects when they speak their native language (Li, Pasquini, et al., 2019). In Li, Pasquini, et al. (2019) we study the functional network of English monolingual subjects and of bilinguals (native in Spanish, bilingual in English). We find that for both groups, when subjects speak their native language the persistent structure is made by BA, WA, preMA, and pre-SMA. Additionally, data in Li, Pasquini, et al. (2019) were acquired for a letter-generation language task, that is, a different task that we use in the present paper (verb generation). Thus, results of Li, Pasquini, et al. (2019) together with the present findings support evidence that the core language network is the most consistent functional architecture beyond intersubject variability and it is not limited to one specific language task.

In terms of functional connectivity, the strongest connectivity weight in the common network ($W_{\text{max}}^{C}$) is between op-BA and preMA ($W_{\text{max}}^{C}=0.74\pm 0.31$, where the average is made across all the subjects that have such link). The triangular BA also connects with the preMA but with about half of the magnitude (*W*^*C*^ = 0.37 ± 0.29). Detailed information on the functional connection of the other areas in the common network is reported in Supporting Information Table S3. Broca’s area has been long recognized as a central language area; its strong connectivity with the preMA(L) is of particular interest since the preMA(L) has been more recently identified as an area with dominant role for language (Duffau et al., 2003). We discuss this result further in the Discussion subsection: Functional and Structural Connectivity of the Common Network.

When we look at the connectivity of the BA subdivisions with Wernicke’s areas, a primary area for language comprehension, we observe that, in the common network, WA only connects to op-BA. This reveals the existence of larger coactivation of the BOLD signal between these two areas that might also be driven by their spatial vicinity (WA is anatomically closer to op-BA than to tri-BA). In more detail, as discussed in Results section, at the individual level we find that WA connects to tri-BA in 15 out of 20 cases whereas it connects to op-BA in 18 out of 20 cases. Therefore, overall, BA subdivisions both connect to WA in several different individuals with a slightly larger presence of WA–op-BA connectivity across subjects. In terms of connectivity weight, when we count only subjects where both op-BA and tri-BA connect to WA, we find that op-BA connects slightly more strongly to WA compared with tri-BA (*W*^*C*^ = 0.17 ± 0.23 versus *W*^*C*^ = 0.15 ± 0.20, respectively).

Furthermore, we observe that op-BA has a larger connectivity than tri-BA both on the number of connections with the rest of the areas in this network (4 versus 3 respectively, the extra one being WA–op-BA) and in terms of functional connectivity weight. Indeed, the average connectivity of the op-BA, across subjects and across areas, in the common network is *W*^*C*^ = 0.45 ± 0.25 (*W*^*C*^ = 0.55 ± 0.20 without the link WA–op-BA), whereas the comprehensive connectivity weight of the tri-BA is *W*^*C*^ = 0.32 ± 0.18.

Finally, we observe that the average values for the common-network functional weights reported in Supporting Information Table S3 have large standard deviations (magnitude comparable with the mean). This result signals a large intersubject variability for the weight of the single functional link across subjects. To investigate this further, we plot the empirical distribution of all the functional links’ weights across subjects and observe that it displays a long tail shape (see Supporting Information Figure S3), which is explicative of the large standard deviation values.

### The Common Network is Part of the Maximum k-core: The Most Resilient Architecture

The notion of *k*-core in theoretical physics has been used as a fundamental measure of centrality and robustness within a network (Morone et al., 2019). Since it was first introduced in social sciences (Seidman, 1983) it has been used in several contexts (Kitsak et al., 2010), as in random network theory (Pittel et al., 1996) or to describe large-scale structure in the brain (Hagmann et al., 2008).

**Figure 4.**
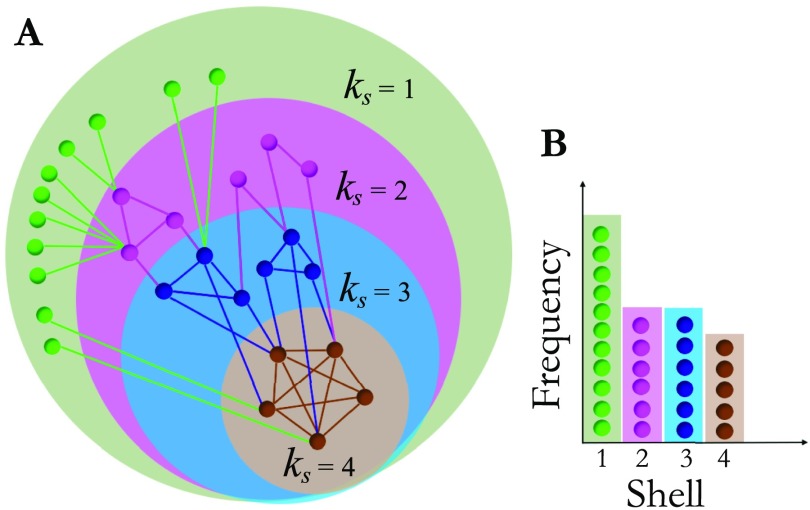
*k*-core and *k*-shell of a network. Panel A illustrates pictorially a network. Nodes in the same disk have the same *k*-core. A *k*-core structure includes subnetworks with higher *k*s, so the 1-core includes the 2-core which, in turn, includes the 3-core, and so forth. Nodes that are in the *k*-core but not in the *k* + 1-core are called *k*-shell and are colored differently. The maximum *k*-core coincides with the maximum *k*-shell, in this network is *k*_core_^max^ = 4 and depicted with brown nodes. Panel B illustrates pictorially the construction of the *k*-core histogram shown in Figure 5. Note that here nodes in each *k*-shell are colored differently, whereas in Figure 5 different colors indicate nodes in different fROIs, piled up according to their *k*-shell as in this panel.

The *k*-core of a given architecture is defined as the maximal subgraph, not necessarily globally connected, made of all nodes having degree (number of connections) at least *k*. In practice, the *k*-core subgraph can be derived by removing from the network all nodes with degree less than *k*. The removal of these nodes reduces the degree of their neighbors, and if the degree of the latter drops below *k* then also these nodes should in turn be removed. The procedure iterates until there are no further nodes that can be pulled out from the network. The remaining graph is the *k*-core of the network. A *k*-core structure includes subnetworks with higher *k*s: *k* + 1, *k* + 2, and so forth. For instance, the 1-core includes the 2-core which, in turn, includes the 3-core and so forth (see Figure 4). In each *k*-core, nodes in the periphery (not included in the *k* + 1-core) are called *k*-shell (*k*_*s*_). Thus, in each network, *k*-core (and *k*-shell) structures are nested within each other with increasing *k*. The innermost structure of the network corresponds to the graph with the maximum *k*-core (Dorogovtsev et al., 2006). Thus, by definition, the max *k*-core is not nested into any other structure with higher *k*. As a consequence, by definition, the max *k*-shell always coincides with the max *k*-core. Figure 4A illustrates *k*-core and *k*-shell structures in a simple explanatory network.

Recently, the maximum *k*-core ($k_{\text{core}}^{\text{max}}$) has been linked to the most resilient structure of biological systems with positive interactions (Morone et al., 2019) and, in an fMRI study of human brains, the $k_{\text{core}}^{\text{max}}$ of the functional connectivity for a visual-task-based experiment has been found to be the most robust structure, which remains active even during subliminal conscious states (subject not aware of seeing images; Lucini et al., 2019).

**Figure 5.**
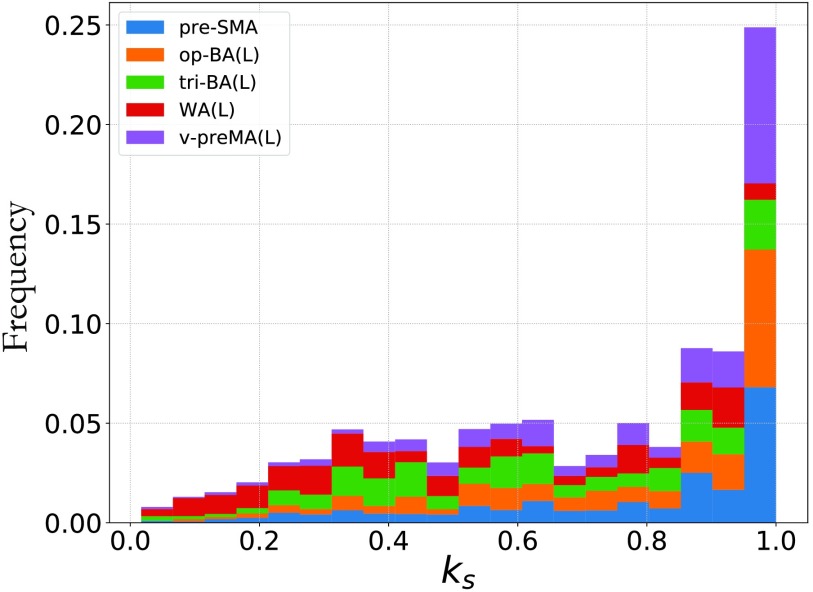
*k*-shell occupancy. The histogram shows the k-shell occupancy for nodes in the four fROIs of the common network of Figure 3. Overall, the majority of the nodes of this structure are located in the maximum *k*-shell, which coincides with $k_{\text{core}}^{\text{max}}$, a quantity linked to the robustness of a complex network (Morone et al., 2019). Of the four fROIs of the common network, the pre-SMA, op-BA, tri-BA, and ventral preMA are mostly part of the $k_{\text{core}}^{\text{max}}$. Wernicke’s area (WA) is more an outlier; it is mostly located in lower *k*-shells and minimally located in the $k_{\text{core}}^{\text{max}}$.

Motivated by these recent findings, we pruned each voxel-level individual functional network until the maximum *k*-core structure, and we investigated to which *k*-core each node (voxel) belongs. We focused on the areas part of the common network (BA, WA, pre-SMA, and preMA) because these are the fROIs that form a persistent language structure across individuals, as shown in Figure 3. We aim to explore whether these regions are part of some significative *k*-core structure that might shed light on the architecture of the network. Our goal is to investigate, across subjects, which fROIs characterize the occupancy of each *k*-shell and, thus, we proceed as follows. For each individual network we compute the *k*-core and *k*-shell of all the nodes (voxels) as described above. Each subject has, in general, a different $k_{\text{core}}^{\text{max}}$(*k*-shell), which is a integer number that can go up to the maximum degree of the subject’s network. Thus, in order to compare results across subjects, we normalize the *k*-core range-of-values to 1, in each subject. We do this by dividing all the *k*-core (*k*-shell) values in each individual network by the individual $k_{\text{core}}^{\text{max}}$ for that network. In Figure 5 we then plot the total *k*-shell occupancy for all the individuals, and we color differently the contribution of each fROI in order to visualize to which *k*-shell they belong.

Results in Figure 5 show that the maximum *k*-shell (which is in turn the maximum *k*-core) is the most populated of all the *k*-shells of the common network. More importantly, if we look at each area individually, we observe that the largest concentration of pre-SMA, op-BA, tri-BA, and v-preMA is in the maximum *k*-shell. Among the areas of the common network, WA is the only area that does not have the largest portion in the $k_{\text{core}}^{\text{max}}$but, rather, populates smaller *k*-shell values. These results are robust to 5−10% variation of the threshold used to build individual functional networks (discussed in the Individual Brain Network Construction section). In order to asses how much of our results are given by construction we compared them with randomized voxel assignation to fROI and recomputed the *k*-shell occupancy as a null model. Results are shown in Supporting Information Figure S4 and show that the relative occupancy of each fROI in the maximum *k*-shell is different from the random case. Furthermore, WA in the real model populates more the smaller shells compared with the null model case. We conclude that our results are not due to a random effect.

In Morone et al. (2019) the authors have shown that, for complex networks with positive couplings, the $k_{\text{core}}^{\text{max}}$of the network is the most resilient structure under decreasing of the coupling weight. In our functional networks, all the links are obtained through thresholding of pairwise correlations that, from our findings, turn out to be all positive. This is because the BOLD signal is extracted from a task-based fMRI experiment, stimulated by an external input. In this way, active voxels are those mostly correlated with the task model and, when computing pairwise correlations among voxels correlated with the same external stimulus, most likely one finds positive correlations, as we observe from our data analysis. This result allows us to interpret the functional networks wired by positive interactions and, therefore, the theory of Morone et al. (2019) applies. Accordingly, we can interpret the maximum *k*-core structure of our network as the most resilient one under decreasing of the correlation weight.

In other words, the circuitry made by the pre-SMA, BA, and preMA represents the most robust structure of the functional network. Wernicke’s area, although it is part of the common network, for the most part does not lay in the $k_{\text{core}}^{\text{max}}$of the network, probably because of its more peripheral anatomical location compared with the other fROIs of this common architecture. Therefore, although it is one of the most important areas for language, it is not part of the most resilient core.

### Rich-Club Response to Changes in Threshold

The rich-club coefficient is a measure of the connectivity as a function of degree (Zhou & Mondragón, 2004). If subnetworks of nodes have a tendency towards a higher percentage of connectivity for larger degrees, then the network is said to be a “rich club.” It has been shown that the brain structural network typically conforms to this paradigm (van den Heuvel & Sporns, 2011). Since the number of connections will naturally increase with degree, it is standard to normalize the rich-club coefficient by the coefficient obtained for a random network with the same number of nodes and degree distribution. The rich-club coefficient is defined as

$$\varphi (k)=\frac{2E_{>k}}{N_{>k}(N_{>k}-1)},$$ (5)

where *E*_>*k*_and *N*_>*k*_is the number of edges and nodes in the network where all nodes with degree less than *k* have been removed. The normalized coefficient is then defined by $\frac{\varphi (k)}{\varphi_{rand}(k)}$ where φ_*rand*_(*k*) refers to the rich-club coefficient calculated in the same network that has been randomly rewired while preserving the degree distribution.

**Figure 6.**
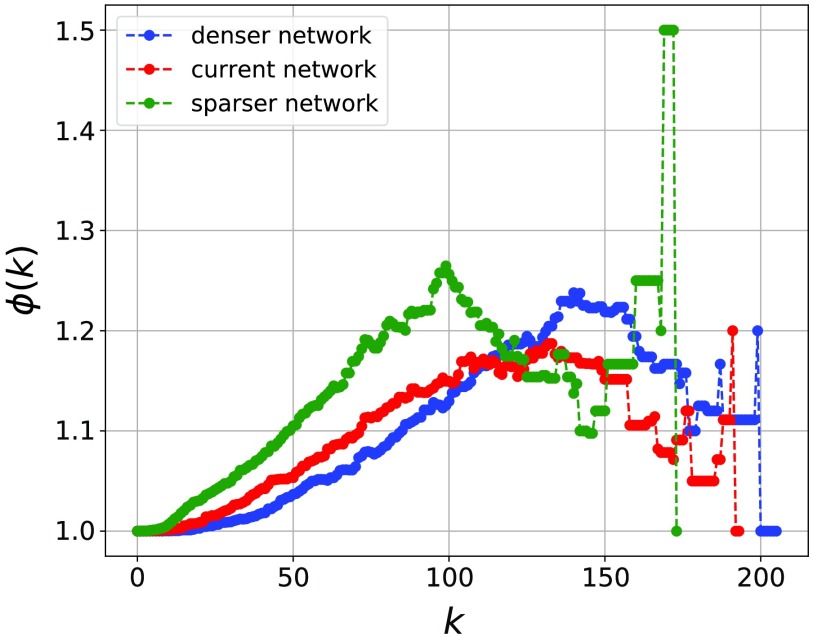
Rich clubs. Normalized rich-club coefficients for a representative subject with 5% (lowered/raised) variation of the current threshold. All other subjects have qualitatively the same normalized rich-club coefficient. The red curve corresponds to the original threshold (network). The blue curve corresponds to a 5% lower variation of the threshold, which leads to a denser network. The green curve corresponds to a 5% higher variation of the threshold, which leads to a sparser network. The figure shows rich-club behavior of the functional brain network, showing an increasing normalized rich-club coefficient.

In the Individual Brain Network Construction section we defined an absolute threshold for the voxel-voxel correlation in order to define what represents a minimum edge weight that defines links in the functional language networks. However, the choice of threshold is somewhat arbitrary and may have an effect on the results. In order to probe how the connectivity responds to changes in the choice of threshold, we show normalized rich-club coefficients for a representative subject in Figure 6.

The threshold was lowered by 5% and raised by 5% in each case, and we can see that the rich-club structure is evident as shown by the rising curve above 1 as a function of *k* up to the high-degree sector of the network where there is high volatility. We also observe that a 5% change in threshold value does not change qualitatively the behavior of the rich-club coefficient. These results show that, in addition to the rich-club behavior found in (van den Heuvel & Sporns, 2011), functional brain networks, such as those we studied in this work, also show the same feature.

## DISCUSSION

In this study, we reconstructed the functional language network of 20 healthy subjects from tb-fMRI data providing information about the functional connectivity between active areas on fMRI maps, with both a voxel- and an fROI-level resolution. The language task designed for the experiment is customarily used in clinical cases and has shown to produce robust activation in previous studies (Brennan et al., 2007; Li, Dong, et al., 2019; Ramsey et al., 2001; Xiong et al., 2000). Functional activation is generally sensitive to the fMRI task employed; our interest in reconstructing functional networks for this specific task aimed to create benchmark results for healthy individuals that can be used as reference for functional networks affected by brain pathologies. Indeed, brain impairments are known to create damage to the functional connectivity. It is therefore paramount to have healthy functional architectures relative to clinical language tasks in order to make the comparison between healthy individuals, and patients’ functional networks possible.

Our main finding is the existence of a common persistent functional network across subjects that wires together BA, WA, ventral preMA, and pre-SMA in the left dominant hemisphere for 17 out of 20 right-handed healthy subjects (see Figure 3). We interpret this circuitry as a core structure for the language task under study since this network persists across nearly all individuals.

Furthermore, we compute the *k*-core of each node (voxel) in the common network—the maximum value of which has been recently linked to network resilience in ecosystems and fMRI studies (Lucini et al., 2019; Morone et al., 2019). We find that three out of four areas of the common architecture (specifically pre-SMA, BA, and preMA) are mostly concentrated in the maximum *k*-core of the network (see Figure 5). This led us to conclude, following the findings of Lucini et al. (2019) and Morone et al. (2019), that these areas are the most robust of the language network in terms of fMRI-correlated signal.

Wernicke’s area is a crucial language area and indeed appears as part of the common network across individuals, yet its type of connectivity with the rest of the fROIs in this architecture is slightly different from the connectivity of the other areas. Indeed, overall, WA shares two connections with other areas in this network, one with BA and one with the preMA, whereas each of the other fROIs has at least three total functional connections. This might be a by-product of the more peripheral location of the WA compared with the other fROIs, which being spatially closer to each other are facilitated to coactivate because of white fibers wiring them together. Wernicke’s area is also the only area among the four in the common network that is not largely part of the maximum *k*-core (see Figure 5). This result is in agreement with the discussion above about this area and, again, might be due to the more perimetric location of the WA in the common network.

Finally, we investigated the functional architecture of the BA anatomical subareas, revealing a different connectivity between tri-BA, op-BA, and the other areas of the common network for this specific language task. In the first subsection, we discuss our findings regarding the functional connectivity of the BA subdivisions, contextualizing them with known white matter connections that these areas share with the rest of the brain, found in other studies.

### Functional and Structural Connectivity of the Common Network

We observe that the left ventral preMA is the most connected area of the common network, with four total connections and the strongest connectivity with op-BA (*W*^*C*^ = 0.74 ± 31) and with pre-SMA (*W*^*C*^ = 0.64 ± 0.31). As shown in Figure 2 for a representative subject, the ventral preMA is functionally connected to all the main cortical language areas of the dominant hemisphere, suggesting that this area may play an important role in speech production (other subjects show qualitatively the same feature; see Supporting Information Figure S1).

Tate, Herbet, Moritz-Gasser, Tate, and Duffau (2014) investigated the crucial cortical epicenters of human language function by means of intraoperative direct cortical stimulation in 165 consecutive patients affected by low-grade glioma. The study shows that speech arrest is localized to the ventral preMA instead of the classical BA. Furthermore, the presence of gliomas growing in the left ventral preMA has been related to a higher percentage of speech deficits than gliomas infiltrating the classical BA, providing a possible clinical correlate of the results of Tate et al. (Bizzi et al., 2012; Tate et al., 2014). Furthermore, Duffau et al. (2003), through intraoperative functional mapping in awake patients, have concluded that the left dominant preMA seems to play a major role in language since its electrical stimulation causes speech disturbances. All these results, together with the findings of our study, pinpoint a central role of the preMA in language production.

However, one must be careful not to overinterpret these results, as the highest connectivity does not necessarily imply a central or essential role of that particular fROI in the network. Using advanced graph theoretical analysis, Morone and Makse (2015) demonstrated that the most connected nodes in a network often do not correspond to the most essential nodes, the elimination of which would lead to collapse of that particular network. This idea has been recently tested on functional networks obtained from fMRI of rodent brains and verified through in vivo pharmaco-genetic intervention (Del Ferraro et al., 2018).

Although the correspondence between structural and functional connectivity is not fully understood yet (Honey et al., 2009), the arrangement displayed by our study is supported by structural evidence. The existence of a physical connection between ventral preMA and BA seems realistic, given their spatial contiguity. Besides representing a shared origin for the main bundles of the dorsal pathway (Chang, Raygor, & Berger, 2015; Dick, Bernal, & Tremblay, 2014), the two areas may be directly connected by a specific opercular-premotor fascicle (described in the next section; Lemaire et al., 2013).

The pre-SMA shows connectivity with both ventral preMA and BA (see Figure 3, Supporting Information Figure S1, and Supporting Information Table S3). These functional connections are consistent with the organization of the structural language connectome to some extent: the frontal aslant tract (FAT), an association motor pathway that underlies verbal fluency and connects pre-SMA and BA (Catani et al., 2013; Ford, McGregor, Case, Crosson, & White, 2010; Jenabi, Peck, Young, Brennan, & Holodny, 2014), likely includes projection to posterior regions of the MFG, corresponding to the ventral preMA (Chang et al., 2015).

The low connectivity weight between ventral preMA and WA (see Supporting Information Table S3) may be explained by the increased distance between the two structures. Of note, we find that the functional connectivity weight between op-BA and WA is similar to that of the ventral preMA and WA (Supporting Information Table S3), which is consistent with their structural connection through the same white matter tract, corresponding to the arcuate component of the AF/SLF system (Chang et al., 2015; Dick et al., 2014).

### Broca’s Area Subdivisions

Our findings show that the subdivisions of Broca’s area present different patterns of connectivity within the language network, with the opercular portion appearing more connected to all the significant nodes of the common network compared with the triangular part. This evidence appears in line with the structural architecture of the network.

The prominent interaction between ventral preMA and op-BA found in this study (*W*^*C*^ = 0.74 ± 0.31, see Supporting Information Table S3) supports the evidence of a structural link between op-BA and preMA, as suggested by Lemaire et al. (2013) using DTI analysis. The authors investigated the structural connectome of the extended BA, identifying the U-shaped opercular-premotor fasciculus that connects the op-BA to the ipsilateral preMA (Lemaire et al., 2013). On the contrary, tri-BA and ventral preMA showed lower functional connectivity (*W*^*C*^ = 0.37 ± 0.29), possibly suggesting indirect communication through the op-BA.

The second strongest functional connection between BA’s subareas and other fROIs of the common network that we find is the link between op-BA–pre-SMA (*W*^*C*^ = 0.35 ± 0.23). These two areas are connected by the FAT (Ford et al., 2010), which originates in the SMA/pre-SMA and terminates into the posteriormost aspect of the inferior frontal gyrus (IFG) (Catani et al., 2013). Triangular BA and pre-SMA share a lower functional connectivity weight (*W*^*C*^ = 0.20 ± 0.21) compared with op-BA and pre-SMA, reflecting the anatomic boundaries of the FAT.

Finally, the functional link between op-BA and WA is in line with the evidence of a dorsal pathway of language between op-BA and STG through the AF/SLF system (dorsal pathway II; Friederici, 2011).

### Limitations of the Study

Functional networks are task dependent and brain impairments, such as brain tumors, affect their functional connectivity (e.g., by destroying functional links or preventing the fMRI activation of entire brain regions). In our study, we focused on a clinical language task since we wanted to enhance a functional network that emerges in clinical studies. We employed healthy subjects because we wanted to find out the network associated with the clinical language task without possible functional damage induced by brain impairments. A comparison with functional networks of patients with brain impairments is of great interest but goes beyond the purpose of the present paper and it is, instead, presented in a follow-up (Del Ferraro, Pasquini, Peck, Holodny, & Makse, 2019). In Del Ferraro et al. (2019) we compare the functional network as well as the structural network (obtained through diffusion tractography imaging) of patients who present speech impairment under awake cortical electrostimulation and patients who do not. As a control, we also compare these networks with the same architecture for healthy subjects, and the results of the present paper are further used as a benchmark.

Our study is limited to a specific clinical language task (i.e., verb generation), and since functional networks are task-dependent, in principle, our results could be limited to this particular language task. In a follow-up work (Li, Pasquini, et al., 2019) we investigate the functional connectivity differences between healthy subjects who are monolinguals, native English speakers, and subjects who are bilingual, native Spanish speakers who speak English as a second language. We employ a different type of language task (i.e., letter generation), and in both datasets we find that the core language network is the same as the one we find in the present paper by using a verb-generation task and discussed in the Results section. This strengthens our findings showing that the core common network is not limited to a specific language task but that it might be a robust structure shared across several tasks. Further studies that make use of other language tasks are needed to elucidate this point and the generality of the core common network.

As a final limitation of our study we remark that the sample size of our data is limited to 20 individuals, and it would be important, in following works, to test the core network analysis on larger datasets.

## ACKNOWLEDGMENTS

We thank Mehrnaz Jenabi for help with AFNI and FSL software and Medeleine Gene for help with mining the data.

## SUPPORTING INFORMATION

Supporting information for this article is available at https://doi.org/10.1162/netn_a_00112. Data that support the findings of this study are publicly available and have been deposited in http://www-levich.engr.ccny.cuny.edu/webpage/hmakse/brain/ (Li, Del Ferraro, et al., 2019).

## AUTHOR CONTRIBUTIONS

Qiongge Li: Formal analysis; Methodology; Software; Visualization; Writing - Original Draft; Writing - Review & Editing. Gino Del Ferraro: Methodology; Software; Supervision; Writing - Original Draft; Writing - Review & Editing. Luca Pasquini: Methodology; Validation; Writing - Review & Editing. Kyung K. Peck: Data curation; Writing - Review & Editing. Hernn A. Makse: Funding acquisition; Investigation; Supervision; Writing - Review & Editing. Andrei I. Holodny: Funding acquisition; Investigation; Supervision; Writing - Review & Editing.

## FUNDING INFORMATION

Hernán A. Makse, National Institutes of Health (http://dx.doi.org/10.13039/100000002), Award ID: 1R01EB022720. Craig Thompson, National Institutes of Health (http://dx.doi.org/10.13039/100000002), Award ID: P30 CA008748. Hernn A. Makse, National Science Foundation (http://dx.doi.org/10.13039/100000001), Award ID: 1515022. Tim Ahles, National Institutes of Health (http://dx.doi.org/10.13039/100000002), Award ID: U54CA137788. Tim Ahles, National Institutes of Health (http://dx.doi.org/10.13039/100000002), Award ID: U54CA132378. Luca Pasquini, Italian Scientists and Scholars in North America Foundation (http://dx.doi.org/10.13039/100009799), Award ID: Imaging chapter award 2018. Luca Pasquini, European Society of Radiology, Award ID: Bracco clinical fellowship 2018. Qiongge Li, City University of New York (http://dx.doi.org/10.13039/100006462), Award ID: Doctoral student research grant.

## TECHNICAL TERMS

Aphasia: Language impairment affecting the production or comprehension of speech and the ability to read or write. Aphasia is caused by brain injuries such as strokes, head traumas, brain tumors, or brain infections.
Language task fMRI: Functional MRI scan where participants perform specific language tasks while the fMRI brain activity is measured. It is used as a radiology clinical routine to determine the localization of language areas in patients with brain impairments.
fROI: Functional region of interest. Group of fMRI active voxels located in the same anatomical brain region that are identified as part of the same active brain area.
Voxel-level functional network: Functional connectivity network where each node represents a voxel and a link depicts the functional interdependency between a pair of voxels.
fROI-level functional network: Course-grained representation of a voxel-level functional network. Each node represents an fROI and a link depicts the functional interdependency between a pair of fROIs.
Direct cortical stimulation: Electrical stimulation of the cerebral cortex with the aim of identifying brain areas that take part in a specific system function (e.g., language, motor). It remains the gold standard for presurgical mapping of the motor cortex and language areas to prevent unnecessary functional damage.
