# Supplementary material for: Core language brain network for fMRI language task used in clinical applications
Source: Netw Neurosci. 2020 Feb 1;4(1):134–54. doi: 10.1162/netn_a_00112 (PMC7006870; doi:10.1162/netn_a_00112)
Supplement: Supplementary file 1 [file netn-04-134-s001.pdf]

Li, Q., Del Ferraro, G., Pasquini, L., Peck., K. K., Makse, H. A., & Holodny, A. I. (2020). Supporting information for "Core language brain network for fMRI-language task used in clinical applications." *Network Neuroscience*, *4*(1), 143-154. https://doi.org/10.1162/netn_a_00112

Supplementary Information

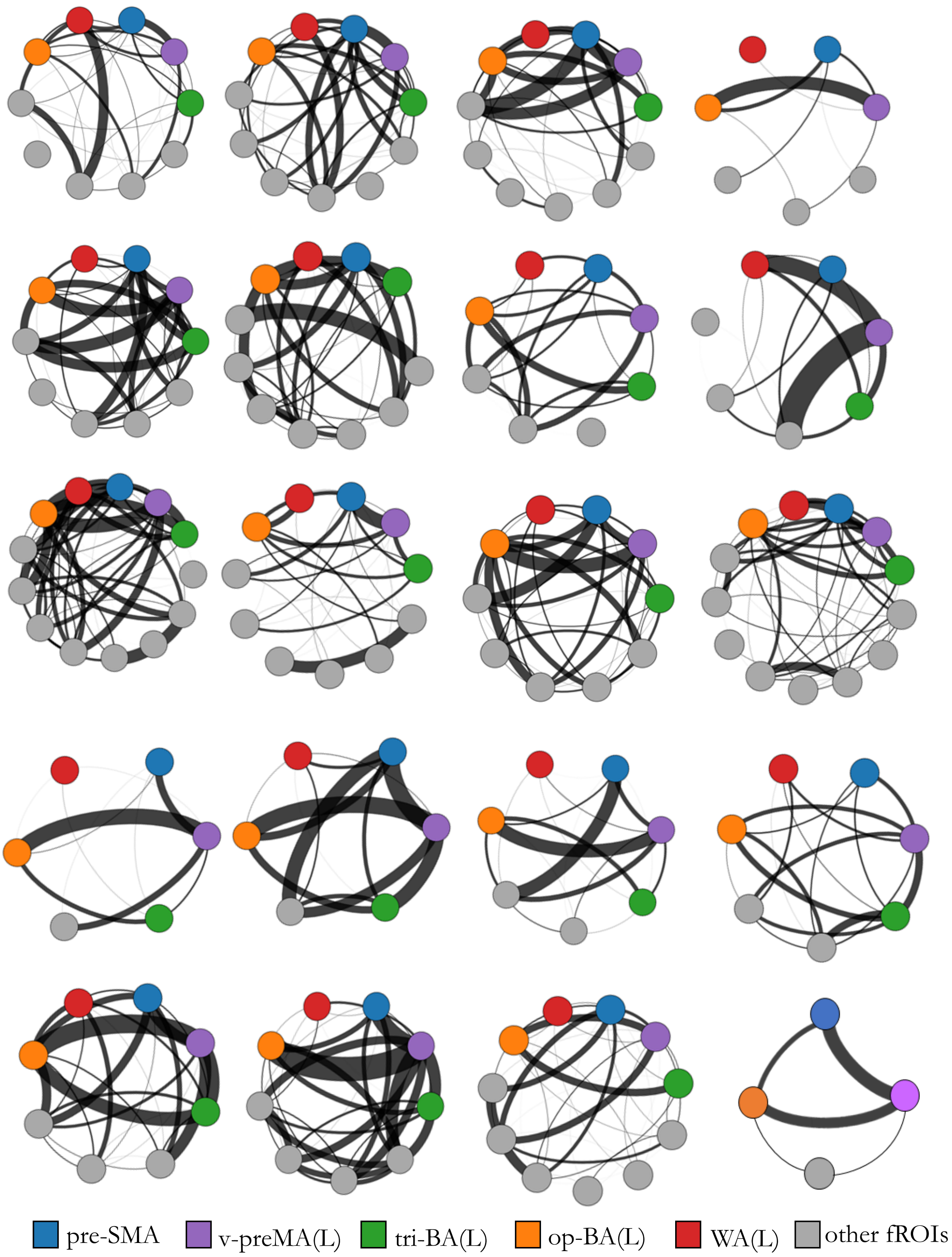

Supplementary Figure S1: **Individual functional network for each subject.** Each node represents a fROIs in the individual network at the fROI-level, links are determined by Eq. (2) and (3) in the main text. Functional ROIs (nodes) which are part of the common network (see Fig. 3) are colored differently according to the legend, all other fMRI active areas are not distinguished and colored in grey. All the nodes (fROIs) are depicted with the same size for illustration purpose and are not proportional to the actual fROI's size. The construction of the functional network for each individual follows the procedure described in individual brain network construction section and in Fig. 2.

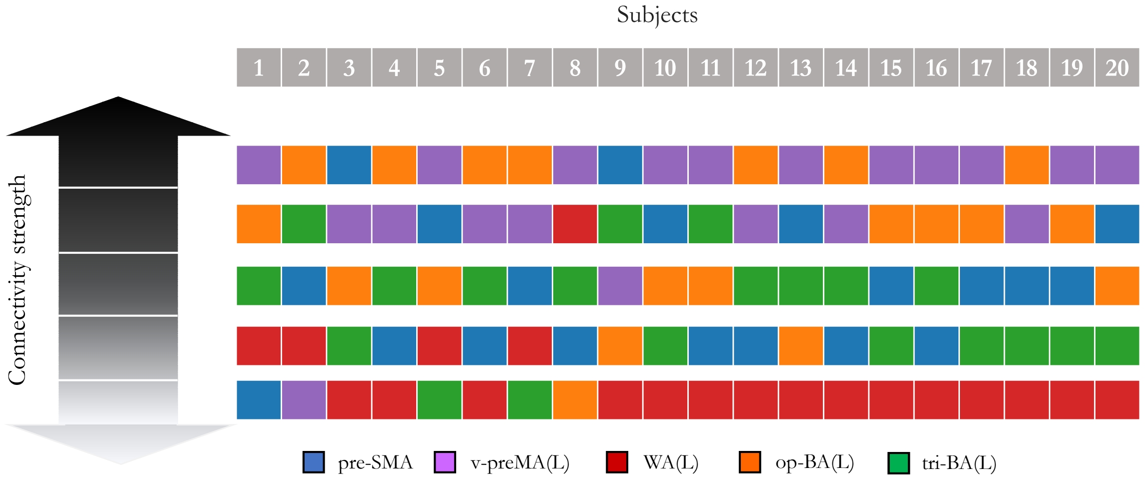

Supplementary Figure S2: **Overall functional connectivity ranking of each fROI.** For each subject the overall functional connectivity strength of each fROI is computed as the sum of all the functional links which, from that fROI, connect to all other fROIs in the common network. For each subjects, fROIs are ranked according to this functional strength. fROIs in the first row at the top are the strongest connected, those at the bottom the least connected. The preMA, in 95% of the cases, is either the most connected or the second most connected area. Differently, WA is either the least connected or the second least connected area in 95% of the cases.

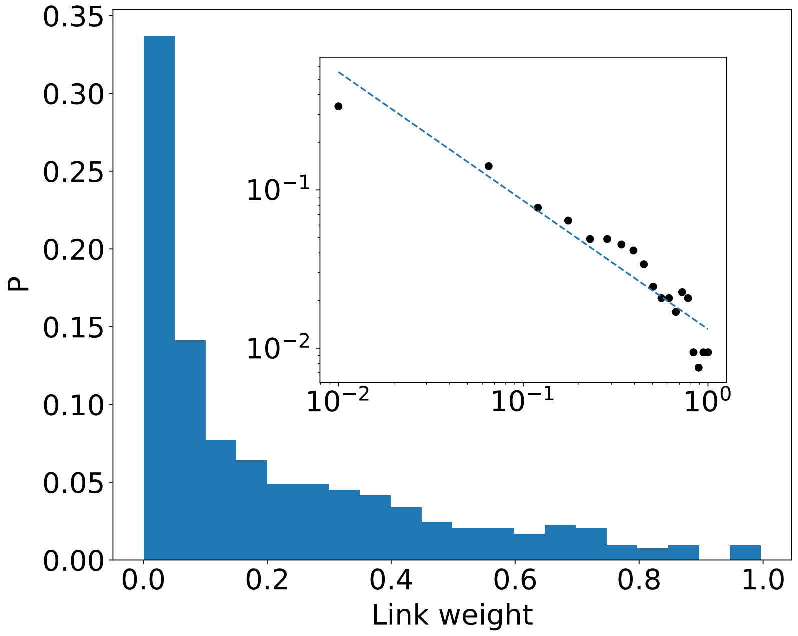

Supplementary Figure S3: **Functional connectivity weight distribution for all the subjects.** Distribution of the functional link's weight across all subjects. The distribution is long tailed and this explains the large variance values in Supplementary Table S3. Inset: same distribution plotted in a log-log scale, data aligns on a straight line which indicates a power-law behaviour for such distribution ($R^2 = 0.85$). The histogram shows all of the normalized link weights for all links in all of the functional networks for all subjects. Each of the link weights in the functional network of a subject is normalized by dividing by the largest value in the individual functional network. This means that there will be a multiplicity of 20 occurrences of a link weight of 1, that is artificial. So we removed that effect. If we hadn't normalized, the peak at unity we would expect to be more spread out over the last few bins is the point.

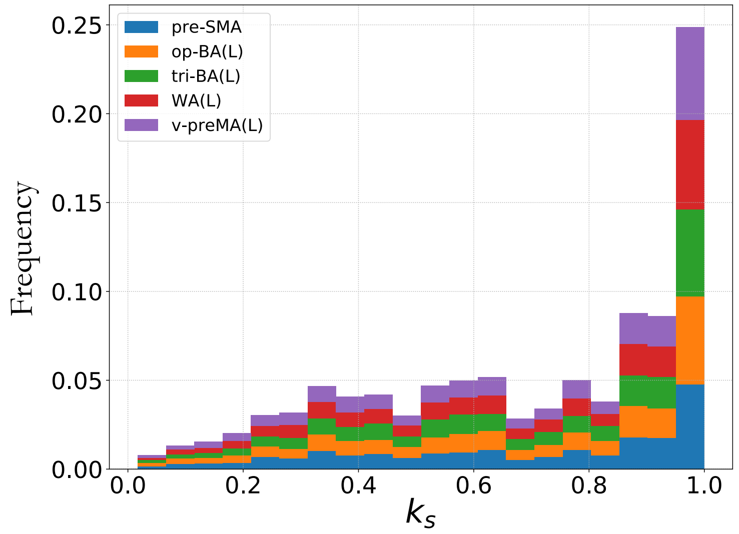

Supplementary Figure S4: ***k*-shell occupancy for the null model.** In order to check whether our results on the maximum k-shell are due to a random effect we compare the results of Fig. 5 discussed in k-core results section with a null model. We generate the null model by shuffling the voxel assignation to fROI in each individual network. The wiring of the network does not change, yet, the fROI label of each module is randomly reassigned. We observe that the relative occupancy of each fROI in the maximum k-shell of the random case is different from the relative occupancy of each fROI in the real model (Fig. 5), so we conclude that our results are not due to a random effect.

Supplementary Table S1: Link's weight between pairs of fROI in each individual network ($\tilde{W}$)

fROI pairs#/ Subject	v-preMA(L) -pre-SMA	v-preMA(L) -WA(L)	v-preMA(L) -op-BA(L)	v-preMA(L) -tri-BA(L)	pre-SMA -tri-BA(L)	pre-SMA -op-BA(L)	WA(L) -op-BA(L)	op-BA(L) -tri-BA(L)
1	0.46	0.01	1	0.68	0.65	0.1	0.24	0.65
2	0	0	0	0	0.19	0.94	0.57	1
3	1	0.14	0.53	0.04	0.14	0.61	0	0.44
4	0.43	0.16	1	0.67	0.14	0.73	0.79	0.71
5	1	0.38	0.36	0.08	0.28	0.1	0.2	0.16
6	0.55	0.31	0.33	0.18	0.08	0.45	0.21	1
7	0.19	0.02	1	0	0.00	0.3	0.02	0
8	0.01	1	0	0.41	0.03	0	0	0
9	1	0.01	0.04	0.13	0.62	0.19	0.09	0.45
10	1	0	0.75	0	0.00	0.38	0	0
11	0.54	0.35	0.58	1	0.23	0.21	0.01	0.76
12	0.58	0.03	1	0.64	0.07	0.35	0.27	0.75
13	1	0.34	0.42	0.42	0.58	0.33	0.07	0.66
14	0.41	0.06	0.97	0.1	0.06	0.19	0.06	1
15	0.47	0.01	1	0.11	0.01	0.04	0	0.29
16	0.34	0.05	1	0.18	0.00	0.13	0.01	0.33
17	1	0.03	0.97	0.82	0.23	0.45	0.02	0.42
18	0.58	0.15	1	0.04	0.01	0.57	0.09	0.35
19	0.7	0.1	1	0.5	0.03	0.22	0.01	0.21
20	1	0.03	0.36	0.33	0.19	0.28	0.43	0.23

Supplementary Table S2: Activated areas across subjects.

Activated area (fROI)	Abbreviations	Activated in # of subjects
Angular Gyrus (L)	AngG (L)	20%
opercularis-Broca's Area (L)	op-BA (L)	95%
triangularis-Broca's Area (L)	tri-BA (L)	90%
Broca's Area (R)	BA (R)	25%
Caudate (L)	Caudate (L)	35%
Caudate (R)	Caudate (R)	25%
Deep Opercular Cortex (L)	DOC (L)	15%
Deep Opercular Cortex (R)	DOC (R)	5%
Middle Frontal Gyrus (L)	MFG (L)	65%
Middle Frontal Gyrus (R)	MFG (R)	10%
pre-Central Gyrus (L)	pre-CG (L)	20%
pre-Central Gyrus (R)	pre-CG (R)	15%
ventral-Premotor Area (L)	v-preMA (L)	95%
dorsal-Premotor Area (L)	d-preMA (L)	50%
Premotor Area (R)	preMA (R)	10%
pre-Supplementary Motor Area	pre-SMA	100%
Supra-Marginal Gyrus (L)	SupMG (L)	60%
Supra-Marginal Gyrus (R)	SupMG (R)	15%
Wernicke's Area (L)	WA (L)	95%
Wernicke's Area (R)	WA (R)	40%

Supplementary Table S3: Links weight between pairs of fROIs in the common network (W^C)

Pair of connected fROIs	Link weights (mean ± stdv)
v-preMA(L) - pre-SMA	0.64 ± 0.31
v-preMA(L) - WA(L)	0.18 ± 0.24
v-preMA(L) - op-BA(L)	0.74 ± 0.31
v-preMA(L) - tri-BA(L)	0.37 ± 0.29
pre-SMA - tri-BA(L)	0.20 ± 0.21
pre-SMA - op-BA(L)	0.35 ± 0.23
WA(L) - op-BA(L)	0.17 ± 0.22
op-BA(L) - tri-BA(L)	0.55 ± 0.28

Supplementary Table S4: fROIs' sizes in each individual network

Subj. #/fROIs	pre-SMA	v-preMA(L)	op-BA(L)	tri-BA(L)	WA(L)
1	55	38	34	0	0
2	72	84	35	30	28
3	88	75	41	41	29
4	144	50	26	45	49
5	72	120	54	36	35
6	69	110	35	42	24
7	96	88	60	27	40
8	93	91	40	71	20
9	79	68	119	20	23
10	57	86	47	30	27
11	160	64	37	26	32
12	17	17	13	23	12
13	21	0	32	20	22
14	15	23	23	11	3
15	18	63	36	24	10
16	54	63	9	8	5
17	71	33	46	5	5
18	49	54	30	0	18
19	47	5	0	36	11
20	47	30	21	33	7
mean ± stdv	184.70 ± 38.43	166.47 ± 31.57	116.21 ± 23.18	90.22 ± 15.47	55.21 ± 12.90

Supplementary Table S5: Number of links between fROI pairs in each individual network

fROI pairs #/ Subject	v-preMA(L) -pre-SMA	v-preMA(L) -WA(L)	v-preMA(L) -op-BA(L)	v-preMA(L) tri-BA(L)	pre-SMA -tri-BA(L)	pre-SMA -op-BA(L)	WA(L) -op-BA(L)	op-BA(L) -tri-BA(L)
1	563	9	1097	967	929	105	216	858
2	0	0	0	0	237	1567	975	1620
3	1564	149	1017	58	148	971	5	623
4	1514	495	4298	2519	257	1718	1582	1848
5	2677	588	597	128	397	152	64	62
6	1248	27	434	51	22	1199	3	1040
7	237	16	1023	0	0	286	11	0
8	19	844	0	867	151	0	0	0
9	2477	13	63	259	1602	412	77	773
10	488	0	284	0	0	177	0	0
11	224	104	184	303	62	59	2	132
12	783	25	960	615	75	369	159	511
13	589	102	97	122	334	172	16	143
14	207	23	446	40	16	64	13	237
15	373	3	643	72	5	17	1	100
16	324	34	757	103	2	107	6	145
17	1456	29	1008	1052	294	473	10	368
18	214	35	472	9	3	283	31	124
19	1085	124	1450	634	27	252	11	176
20	855	12	137	113	138	210	114	55
